# Implication of the knowledge and perceptions of veterinary students of antimicrobial resistance for future prescription of antimicrobials in animal health, South Africa

**DOI:** 10.4102/jsava.v90i0.1765

**Published:** 2019-10-17

**Authors:** Peter W. Smith, Michael Agbaje, Lerica LeRoux-Pullen, Deborah van Dyk, Legesse K. Debusho, Aminu Shittu, Mohamed M. Sirdar, Olubunmi G. Fasanmi, Oluwawemimo Adebowale, Folorunso O. Fasina

**Affiliations:** 1 Department of Production Animal Studies, University of Pretoria, Pretoria, South Africa; 2 Department of Veterinary Microbiology and Parasitology, College of Veterinary Medicine, Federal University of Agriculture, Abeokuta, Nigeria; 3 Department of Paraclinical Sciences, University of Pretoria, Pretoria, South Africa; 4 Department of Statistics, College of Science, Engineering and Technology, University of South Africa, Johannesburg, South Africa; 5 Department of Public Health and Preventive Medicine, Faculty of Veterinary Medicine, Usmanu Danfodiyo University, Sokoto, Nigeria; 6 Onderstepoort Veterinary Research, Agricultural Research Council, Pretoria, South Africa; 7 Department of Animal Health, Federal College of Animal Health and Production Technology, Ibadan, Nigeria; 8 Department of Veterinary Public Health and Reproduction, College of Veterinary Medicine, Federal University of Agriculture, Abeokuta, Nigeria; 9 Emergency Centre for Transboundary Animal Diseases, Food and Agriculture Organisation of the United Nations, Dar es Salaam, United Republic of Tanzania; 10 Department of Veterinary Tropical Disease, University of Pretoria, Pretoria, South Africa

**Keywords:** antimicrobials, stewardship, training, undergraduate students, perception, practice

## Abstract

Understanding the knowledge and perceptions of veterinary students of antimicrobial resistance (AMR) as potential future prescribers of antimicrobials may serve as an opportunity to improve stewardship of AMR. Pre-final (*n* = 42) and final (*n* = 29) year veterinary students of the University of Pretoria completed questionnaires to determine their knowledge and perceptions of AMR. Of the 71 respondents, mixed practice (48%) and small animal practice (45%) were the most preferred career choices post-graduation, with the field of gross pathology being the least preferred. Over 80% of the respondents believed that veterinary practitioners’ misuse of antimicrobials contributes to AMR and a higher percentage (98.6%) believed that farmers’ misuse of antimicrobials encourages the development of AMR, in particular, in food animals (60.6%) compared to companion animals (50.7%). Agreement in the ranking of abuse of antimicrobials between pre-final and final year students was fair (36.4%; kappa 0.3), and the most abused antimicrobials in descending order listed by the students were tetracyclines, penicillins, sulphonamides and aminoglycosides. There was wide disparity between training and potential field application, as well as variations in the correct matching of antimicrobials to their respective antibiotic classes. Responses to the clinical application of antimicrobials also varied widely. Despite the apparent teaching of AMR to veterinary students, gaps may exist in the translation of theoretical concepts to clinical applications, hence the need for focused and targeted antimicrobial prescription and stewardship training to bridge these potential identified gaps.

## Introduction

Antimicrobial resistance (AMR) is a spreading and important public health concern globally (World Health Organization [WHO] 2001). In the tropics and particularly in many developing economies, the burden of animal and human infectious diseases is enormous. Coupled with weak veterinary infrastructures in many countries, deficient effective consultation and treatment, the inability to afford expensive drugs and the burden of counterfeit drugs have added to the disease burdens (Newton, Green & Fernández 2010; Schelling et al. 2005). To date, antimicrobials remain one of the most abused, overused and misused categories of medicine in the field of veterinary medicine and public health, and these have encouraged the development of AMR (Mehrotra, Dougherty & Poppe 2003). Between 2002 and 2004, the South African animal industry utilised 1 054 177 kg of in-feed, 190 400 kg of water-based and 269 794 kg of parenterally administered antimicrobials (Eagar, Swan & Van Vuuren 2012) and the trend is foreseen to increase with more intensification in the industry. In 2014, 2015 and 2016, a total of 23 333 808 kg, 23 857 579 kg and 22 205 520 kg of antibiotics were reportedly imported for use in the South African medical industry, veterinary industry and the private sector, respectively, while the public sector utilised 1 670 911 057 kg, 1 553 790 791 kg and 1 906 200 029 kg during the same period (Schellack et al. 2017). The resultant increase in the prevalence of antimicrobial-resistant organisms has influenced national and international efforts towards combating AMR development through planned reduction programmes for the use of antimicrobials (Harbarth et al. 2015). Currently, based on the efforts made globally, significant gains have been made, including but not limited to the use and misuse of antimicrobial agents as growth promoters: At least 110 of the 155 countries that responded to the questionnaires (71%) did not use any antimicrobial agents for growth promotion in animals in 2017, either with or without legislation or regulations. However, 45 countries (29%) still use antimicrobials for growth promotion, with regulations (18/45; 40%) and without a regulatory framework (27/45; 60%) (World Organisation for Animal Health 2018). Such efforts have included but are not limited to the global (and national) action plans and policies on AMR: the Global Antibiotic Resistance Partnership (GARP), Alliance for the Prudent Use of Antibiotics, European Antibiotic Awareness Day, Canadian Antimicrobial Resistance Alliance, European Antimicrobial Resistance Surveillance Network (EARS-Net), Joint Programming Initiative on Antimicrobial Resistance, Center for Disease Dynamics, Economics & Policy-GARP (CDDEP-GARP) and National Antimicrobial Resistance Monitoring System, among others. In South Africa, the South African Antimicrobial Resistance Strategic Framework has been produced, covering the period 2018–2024 to guide national actions on reducing the burden of AMR (Department of Health [DoH]; Department of Agriculture, Forestry and Fisheries [DAFF] 2018).

In addition, increased attention has been focused on educating healthcare workers, and veterinary and medical students on the rational prescription of antimicrobials (antimicrobial stewardship) as a measure to reduce the burden of AMR (WHO 2012). While the medical, veterinary and other health professional curricula have been enhanced in developed countries (Abbo et al. 2013; Castro-Sanchez et al. 2016; Davenport, Davey & Ker 2005; Guardabassi & Prescott 2015; Heaton, Webb & Maxwell 2008; O’Shaughnessy et al. 2010; Ross & Maxwell 2012), such improvements in the developing economies seem to be lacking or non-reported. However, the training methods and contents of courses in the South African veterinary faculty are deemed adequate, but in view of the issues above, this study was aimed at assessing the knowledge and perceptions of veterinary students on AMR as future antimicrobial prescribers. It is expected that the outcome of the study will help review how the retention of knowledge can be enhanced to promote better antimicrobial stewardship.

## Materials and methods

### Study design

This is a quantitative study designed to explore the level of knowledge (and retention thereof), and perception of AMR using questionnaires among pre-final and final year students at the Faculty of Veterinary Science, University of Pretoria. A standardised questionnaire was prepared and jointly reviewed by specialist veterinarians (a pharmacologist, poultry specialist, pig specialist, production animal specialist and microbiologist, all from the University of Pretoria). The questionnaire was pre-tested among 15 selected second-year veterinary students and adjusted appropriately (online Appendix 1).

### Recruitment and selection of candidates

The study was conducted with 240 (total number of pre-final and final year) students of Veterinary Science at the Faculty of Veterinary Science, University of Pretoria (the only faculty offering Veterinary Science in South Africa) at the end of the 2014 academic session. Participation was voluntary and each student was allowed to withdraw during the course of the study. The inclusion criteria were for the students to have had the veterinary pharmacology and microbiology training during the preclinical or paraclinical years of veterinary training. Instructions were available to guide responses to the questionnaire, but each student was instructed to complete the questionnaire independently to prevent undue influence that may bias the outcome. Briefly, the degree of Bachelor of Veterinary Science (BVSc) in Pretoria recently underwent a revision from the old curriculum (BSc [veterinary biology] + BVSc, totalling 7 years). Basically, the newly admitted students spend the first year in the preliminary science courses and start a professional programme in the faculty from year 2. Courses include Medical Terminology, Animal Nutrition, Introductory Animal Production, Veterinary Professional Life and courses aimed at soft skills development as well as ethical and professional aspects of the veterinary profession.

From the second year of study, basic veterinary disciplines in Anatomy, Histology, Microbiology and Physiology as well as modules in Animal Science, Pasture Science and Professional Life are taught. Courses in Infectious Diseases, Parasitology, Toxicology, Pharmacology, Organ Pathology and Professional Life are the main focus of year 3 to train the students in the causes and effects of disease. The students are exposed to diagnostics and therapeutics in the fourth year and the first semester of the fifth year and community engagement is also integrated. In the second semester of the fifth year, the emphasis primarily centres on the didactic components of the elective modules chosen by students. In the final (sixth) year of the study, students receive experiential training in the core and elective components in the academic hospital and in satellite and other approved facilities. These final year components enforce frequent revisiting of materials and training received in the earlier years of study.

### Data analysis

Eleven antimicrobial agents were ranked according to their degree of abuse based on the students’ perceptions of these agents. Frequencies of assigned ranks (score 1–11) were determined and entered into a Microsoft Excel spreadsheet (Redmond, WA, United States [US]). The score ranking of each antibiotic was determined as the antimicrobial with the highest students’ perception frequency for that score; and where two antimicrobial agents had the same frequency, they were ranked equally and assigned the same score.

### Statistics

Statistical analyses were performed on the Microsoft Excel spreadsheet (Redmond, Washington, US) and Stata version 9.0 (Stata Corporation, Lakeway Drive, College Station, Texas, US). Data were presented as mean, percentages and proportions with 95% confidence limits. Continuous data between pre-final and final year students were compared by a two-sample *t*-test, while categorical data were analysed using the Spearman (Rho) rank order statistical tool. Analysis was considered significant when *p* ≤ 0.05.

### Ethical considerations

As no invasive procedure was requested and no sampling of any human or animal was required, no specific ethical approval was obtained. However, each student signed the consent form to participate and reserved the right to withdraw during any part of the study. Approvals to conduct the study were granted through the project number 2013/05 (Open Access Learning in Stewardship of Antimicrobials) and the 2013/2014 SA Department of Higher Education and Training (DHET) Scholarship of Teaching and Learning (perception of veterinary students in South Africa on antimicrobial administration in intensive animal production).

## Results

### Demographics

Only 109 of the 240 questionnaires handed out to students were returned (response rate: 45%) with only 71 (29.6%) consisting of 42 (59.15%) pre-final and 29 (40.84%) final year students deemed appropriate for further analysis. Of the participating respondents, 52 (73.2%) were women, while only 19 (26.8%) were men (Table 1).

**TABLE 1:** Descriptive statistics and career choices of pre-final year and final year veterinary students, Faculty of Veterinary Science, University of Pretoria, Onderstepoort, South Africa.

Variable (*n* = 71)	Category	Number	%	95% confidence interval
Gender	Male	19	26.8	16.2; 37.3
	Female	52	73.2	62.7; 83.8
Class	Final year	29	40.8	29.1; 52.6
	Pre-final year	42	59.1	47.4; 70.9
Likely career choice post-graduation†	Small animal practice	32	45.1	33.8; 56.7
	Equine practice	13	18.3	10.6; 28.6
	Mixed practice	34	47.9	36.5; 59.5
	Feedlot	6	8.5	3.5; 16.8
	Dairy	8	11.3	5.4; 20.3
	Wildlife	19	26.8	17.5; 37.9
	Gross pathology	0		0
	Pharmaceutical industry	3	4.2	1.1; 11.1
	State service	8	11.3	5.4; 20.3
	Beef cattle	15	21.1	12.8; 31.8
	Sheep and goats	11	15.5	8.4; 25.3
	Pig	7	9.9	4.4; 18.5
	Poultry	7	9.9	4.4; 18.5
	Laboratory medicine or clinical pathology	2	2.8	0.5; 9.0
	Exotic pet medicine	11	15.5	8.4; 25.3
	Education	3	4.2	1.1; 11.1
	Undecided	6	8.5	3.5; 16.8
	Other choices	5	7.0	2.6; 14.9
Previous knowledge in the field‡	No	64	90.1	83.0; 97.2
	Yes	7	9.9	2.8; 17.0

† , The mean ± standard deviation for number of career choice is 3 ± 2; (Minimum = 1; Median = 2; Maximum = 12; one person made no choice) and the mean ± standard deviation for age of all respondents was 25.2 ± 3.1 years (Minimum = 22; Median = 25; Maximum = 41 years).

‡ , Previous knowledge in the field means the student has done previous studies at post-secondary school levels in pharmacology, biological research, microbiology or pharmacy, which may bias the opinion of the respondent or influence the responses to antimicrobial-related questions.

Mixed practice (48%) and small animal practice (45%) were the most preferred career choices post-graduation, with gross pathology being the least preferred. Pharmaceutical industry, laboratory medicine or clinical pathology and education fields were minimally subscribed, while six (8.5%) were undecided and five (7.0%) subscribed to other choices (Table 1). Previous knowledge may have influenced or biased the responses of 10% of the students with previous degrees in pharmacology, biological research, microbiology or pharmacy (data not shown, Table 1).

### Students’ perception of antimicrobial abuse and misuse

Students’ perception of antimicrobial prescription, stewardship, misuse and education are summarised in Table 2. All respondents mentioned that AMR is an increasing threat to humans and animals. Over 80% of the students believed that veterinary practitioners’ misuse of antimicrobials contributes to AMR and a higher percentage (98.6%) believed that farmers’ misuse of antimicrobials encourages the development of AMR, in particular in food-producing animals (60.6%) compared to companion animals (50.7%). However, a significant difference (*p* = 0.02) existed between pre-final and final year students on the question: ‘The use of antimicrobials in the food-producing animal industry contributes more to AMR than their use in companion animals’. Also, there was a difference (*p* = 0.01) between pre-final and final year students in observed evidence of misuse of antimicrobials in students’ training facilities.

**TABLE 2:** Perception of antimicrobials of all students who agreed or strongly agreed to the questions.

Variable	All % (*n* = 70)	Pre-final % (*n* = 42)	Final % (*n* = 28)	p*
Antimicrobial resistance is an increasing global threat to human and animal health	100.0	100.0	100.0	1.00
The misuse of antimicrobials by veterinary practitioners contributes significantly to antimicrobial resistance	84.5	81.0	89.3	0.35
The misuse of antimicrobials by farmers contributes significantly to antimicrobial resistance	98.6	97.6	100.0	0.41
The inappropriate use of antimicrobials in food-producing animals significantly contributes to antimicrobial resistance in human pathogens	60.6	59.5	60.7	0.92
The inappropriate prescription of antimicrobials by human medical doctors is the main contributor to antimicrobial resistance in human pathogens	94.4	95.2	92.9	0.68
I have received formal lectures on the rational use of antimicrobials during my undergraduate training	98.6	97.6	100.0	0.41
My undergraduate training has prepared me well for making informed decisions when choosing an ideal antimicrobial for an individual patient	76.1	73.8	78.6	0.65
As an individual in practice, I can significantly contribute to preventing an increase in antimicrobial resistance	84.5	85.7	85.7	1.00
The misuse of antimicrobials was evident in the facilities where I have trained	23.9	11.9	39.3	**0.01**
Governing bodies in Africa are doing enough to help prevent a rise in antimicrobial resistance	1.4	0.0	3.6	0.22
Educating laypeople on the importance of antimicrobials as controlled scheduled compounds will have a positive effect on decreasing the rise in antimicrobial resistance	87.3	88.1	85.7	0.77
The use of antimicrobials in the food-producing animal industry (farm animals) contributes more to antimicrobial resistance than their use in companion animals	50.7	38.1	67.9	**0.02**
Banning the use of prophylactic antimicrobials in food-producing animals will have a negative effect on animal welfare	45.1	42.9	50.0	0.56
Banning the use of prophylactic antimicrobials in food-producing animals will have a positive effect on decreasing the rise in antimicrobial resistance	56.3	52.4	60.7	0.49
Banning the use of antimicrobials as growth promoters in food-producing animals will have a positive effect on decreasing the rise in antimicrobial resistance	57.7	61.9	50.0	0.33
Improved use of vaccines, biosecurity measures and hygiene will decrease the need for antimicrobials in the food-producing industry	95.8	95.2	96.4	0.81
Adhering to meat and milk withdrawal periods will help decrease the rise in antimicrobial resistance in human pathogens	67.6	64.3	71.4	0.54
Broad-spectrum antimicrobials are ideal to use as first-line antimicrobials	38.0	35.7	42.9	0.55
Third and fourth generation antimicrobials should only be used as a last resort in treatment	87.3	83.3	96.4	0.10
Long-acting antimicrobials are more ideal for use in food-producing animals than shorter-acting equivalents	21.1	19.0	21.4	0.81
Cultures and antibiotic sensitivity testing, for example, antibiograms, should be performed as frequently as possible to guide antimicrobial use	97.1	95.2	100.0	0.25
Financial constraints of animal owners in Africa disallow the use of cultures and antibiotic sensitivity testing, for example, antibiograms during an infection	77.5	76.2	78.6	0.82
Drug legislation in Africa is on par with legislation in the rest of the world	18.3	14.3	21.4	0.44
I am confident that new classes of antimicrobials will be available in the near future to solve current resistance problems	7.0	4.8	10.7	0.34
The choice of an antimicrobial(s) by a veterinarian should largely be determined based on the cost implications to the farmer	19.7	21.4	17.9	0.72
I am confident in my ability to choose the ideal antimicrobial agents for a specific patient or group of animals to ensure optimal efficacy and safety	39.4	28.6	57.1	**0.02**

Note: Data in bold indicate the significant difference between groups.

* , Two-sample *t*-test for proportions was conducted to assess whether there is a significant difference between the responses of pre-final and final year students.

More than 95% of respondents believed that improvements in vaccine usage, biosecurity measures and hygiene will limit the need for antimicrobials in food animals, while over 60% of participating students perceived that an adherence to meat and milk withdrawal periods will mitigate the development of AMR. Furthermore, over 55% of the students believed that a ban on the use of antimicrobials as prophylactics and as growth promoters in food animal practice will reduce the increase in AMR development.

### Antimicrobial education

Impressively, South African veterinary students expressed confidence in their overall knowledge of antimicrobials (mean score of 1.8 ± 0.8), especially with regard to differentiating between time- and concentration-dependent antimicrobials (*p* ≤ 0.0001), interpreting the antibiogram (*p* ≤ 0.0001) and choosing the most ideal route for administering a specific antimicrobial (*p* ≤ 0.04) (Table 3). Conversely, student responders were significantly unsure, vague or had no idea of: (1) the spectrum, effect, distribution, indications, side effects and contraindications of the most commonly used antimicrobial classes in veterinary medicine, (2) resistance mechanisms, (3) choosing the desired time frame for therapy, (4) choosing an alternative if the first choice of antimicrobial therapy failed and (5) the design of an integrated treatment protocol for a specific animal with an infection which includes supportive therapy (Table 3).

**TABLE 3:** Perceived knowledge of antimicrobials of all participating (pre-final and final year) veterinary students (*n* = 70), Faculty of Veterinary Science, Onderstepoort.

Variable	Median score	Mean score ± s.d. (all students)	Confident (%)	Unsure (%)	Vague (%)	No idea (%)	*p*
Spectrum, effect, distribution, indications, side effects and contraindications of the most commonly used antimicrobial classes in veterinary medicine, as well as the implication thereof	2	2.0 ± 0.6	17.1	62.9	20.0	0.0	< 0.0001
The difference between time-dependent and concentration-dependent antimicrobials	1	1.5 ± 0.7	**65.7**	21.4	12.9	0.0	< 0.0001
Resistance mechanisms	2	2.0 ± 0.9	32.9	34.3	30.0	2.8	< 0.0001
Making a Gram-stain	1	1.7 ± 0.9	55.7	18.6	21.4	4.3	0.1800
Interpreting antibiograms	1	1.6 ± 0.8	**62.9**	21.4	12.9	2.8	0.0020
Finding reliable sources of information to guide empirical use of antimicrobials	2	1.7 ± 0.8	50.0	34.3	14.3	1.4	1.0000
Choosing the most ideal route for administering a specific antimicrobial	1	1.6 ± 0.8	**58.6**	30.0	8.6	2.8	0.0400
Choosing the desired time frame for (duration of) therapy	2	1.9 ± 0.9	37.1	42.9	10.0	10.0	0.0020
Choosing an alternative if my first choice of antimicrobial therapy failed	2	2.0 ± 0.8	30.0	47.1	18.6	4.3	< 0.0001
Designing an integrated treatment protocol for a specific animal with an infection which includes supportive therapy	2	2.1 ± 0.9	24.3	50.0	18.6	7.1	< 0.0001
**Total**	**-**	**1.8 ± 0.8**	**-**	**-**	**-**	**-**	**-**

Note: Data in bold indicate significant difference between the confident group and other responses.

The mean score can range from 1 (confident) to 4 (no idea). The closer to 1 a score is, the more confident the students were about their knowledge of the question. The *p*-value represents the difference between students who were confident of their knowledge of the questions and those who were unsure, vague or had no idea pulled together as a single category.

s.d., standard deviation; No., number.

**FIGURE 1:**
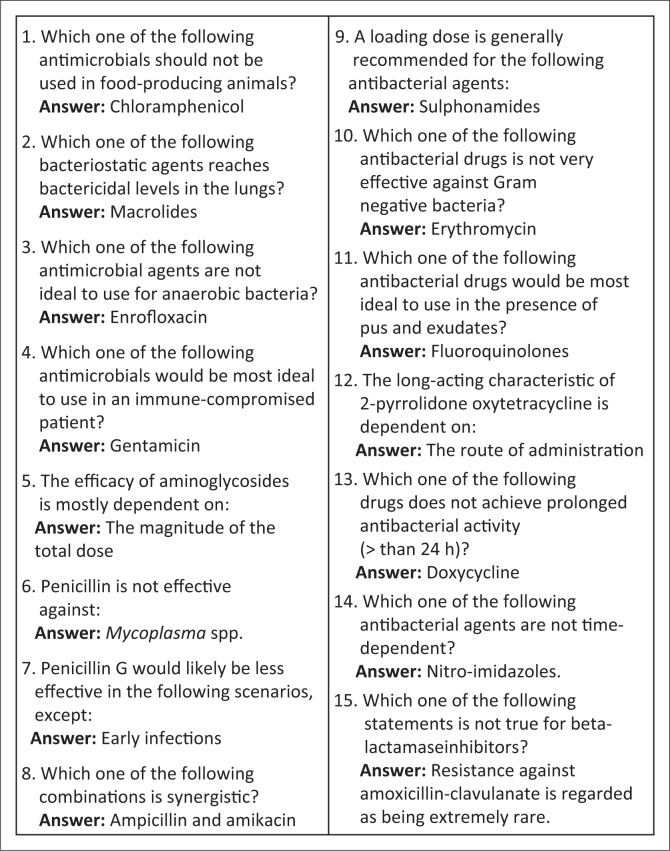
Specific questions on characteristics of individual antimicrobial agents (*n* = 71).

Generally, the agreement in ranking of the degree of abuse of antimicrobials based on students’ perception between pre-final and final year students was fair (36.4%; kappa 0.3). Tetracyclines, penicillins, sulphonamides and aminoglycosides were considered the most abused antimicrobials in descending order, while the polypeptides, cephalosporins and formulations of combined antimicrobials were the least abused (Table 4). There was a wide variation (range: 38% – 96%) in the correct matching of individual antibiotics with their corresponding antibiotics class, with correct matching of penicillin (39.4%) and macrolide (38.0%) groups being the lowest, while peptide antibiotics (95.8%), aminoglycosides (94.4%) and beta-lactams (80.3%) were the medicines most frequently correctly matched (Table 5). Furthermore, correct responses to specific questions on the clinical application of antimicrobials varied widely (range 15.5% – 73.2%, Figure 1).

**TABLE 4:** Ranking of the degree of abuse of antimicrobials based on students’ perceptions, Faculty of Veterinary Science, Onderstepoort.

Antimicrobials	Ranking of abuse of antimicrobials		
	All (*n* = 70)	Pre-final year (*n* = 42)	Final year (*n* = 28)
Tetracyclines	1st	1st	1st
Penicillins	2nd	2nd	2nd
Sulphonamides	3rd	3rd	3rd
Aminoglycosides	4th	6th	4th
Amphenicols	5th	5th	8th
Macrolides	6th	4th	6th
Quinolones	7th	7th	10th
Polypeptides	8th	8th	9th
Cephalosporins	9th	9th	5th
Combiotics	10th	10th	7th
Others	11th	11th	11th

Note: Spearman (Rho) rank order correlation coefficient (*r*_*s*_) = 0.76; *p*-value < 0.01.

**TABLE 5:** Matching of specific antimicrobials with their class (*n* = 71).

Variable	No. of correct responses	Percentage ± s.d.	CI _95_%	No. of wrong responses	%
Beta-lactams	57	80.3	69.5; 88.0	14	19.7
Penicillins	28	39.4	28.9; 51.1	43	60.6
Cephalosporins	44	62.0	50.3; 72.4	27	38.0
Tetracyclines	60	84.5	74.2; 91.3	11	15.5
Aminoglycosides	67	94.4	86.0; 98.2	4	5.6
Macrolides	27	38.0	27.6; 49.7	44	62.0
Amphenicols	44	62.0	50.3; 72.4	27	38.0
Fluoroquinolones	55	77.5	66.4; 85.7	16	22.5
Sulphonamides	45	63.4	51.7; 73.7	26	36.6
Peptide antibiotics	68	95.8	87.8; 99.0	3	4.2
**Total mean knowledge score**		**69.7 ± 20.5**	**55.1; 84.4**	**-**	**30.3**

Note: Total correct matching score of 69.7% was obtained for all the surveyed students (*n* = 71). Penicillin and macrolide groups have the two worst matching scores of 39.4% and 38.0%, respectively. A significant majority were able to match peptide antibiotics (95.8%), aminoglycosides (94.4%) and beta-lactams (80.3%) most correctly.

s.d., standard deviation; No., number.

## Discussion

The rapidity with which AMR is developing and spreading in animals has attained a public health emergency or epidemic proportion as agreed by all participants in this study. Most respondents intended to pursue a post-graduation career in mixed and small animal practices, thereby suggesting a high likelihood that they will be responsible for prescribing antimicrobial agents in the future. However, a significant gap existed between their current levels of knowledge and the knowledge required for the prescription of antimicrobials. It should be understood that these were veterinary clinicians in training and the degree of uncertainties at this stage may not be completely negative. It has been known that the confidence of a professional ultimately grows post-graduation as more practice is undertaken. In addition, the outcome of this study should be taken with a certain degree of caution because final year students have an additional advantage of reinforced training and more extensive clinical experience compared to the pre-final year students. Furthermore, the different degrees of practical exposure between fifth and final year students may also have influenced the overall scores obtained in the assessment.

The belief that the misuse of antimicrobials contributes to the rising scourge of AMR, particularly in the food animal sector, is not unexpected based on the level of exposure of these students to clinical rotations. Aside from therapeutic applications, where antimicrobials have been copiously applied in food animals as prophylactics and growth promoters, there have been linkages with the development of resistance in certain strains of bacteria (Stege, Jacobsen & Thougaard 2003; South African National Veterinary Surveillance and Monitoring Programme for Resistance to Antimicrobial Drugs [SANVAD] 2007; White et al. 2001; Witte 2000). This suggests the need for a critical review of guidelines for antimicrobial prescription in training facilities as well stricter measures against needless antimicrobial prescriptions and use (Arnold & Straus 2009). In addition, supervising clinicians should clearly explain to students on clinical rotations the basis for taking decisions on the appropriateness of use of antibiotics in the management of health conditions. It should be noted that from the time of the SANVAD report (2007) until now, the regulatory authorities in South Africa have made efforts to regulate more strictly and reduce the use and availability of in-feed medication for prophylaxis or growth promotion. This should positively influence the burden of resistance organisms in South Africa.

Nearly all the respondents believed in alternative methods of reducing the burden of AMR (improved vaccination, biosecurity measures and adherence to general hygiene practices). Evidence exists that vaccine usage and adoption of biosecurity measures may prevent or reduce exposure to disease pathogens (Barrow & Wallis 2000; Dial et al. 1992; Jansen & Anderson 2018; Klugman & Black 2018), and in a recent report by the Interagency Coordination Group on AMR, issues raised above have strongly been advocated for (Davies & Wray 1997; Fedorka-Cray, Harris & Whipp 1997; IACG 2019).

Furthermore, approximately 50% of the students in this study held the opinion that strict adherence to meat and milk withdrawal periods and a ban on the use of antimicrobials as prophylactic medicines or growth promoters may reduce the burden of AMR. However, these effects may vary with antimicrobial type. For example, an earlier study observed increased tetracycline resistance in enterococcal bacteria, but not the same in resistance to erythromycin despite increased usage of both antimicrobials (Danish Integrated Antimicrobial Resistance Monitoring and Research Programme [DANMAP] 2005). Although 21% of the students agreed that long-acting antimicrobials are suitable in food-producing animals, it appeared that the large majority were unaware that these medications were often used in the feedlot system and in other production systems to improve feed efficiency and prevent coccidiosis. In a country like Australia, efforts have been intensified to reduce the use of antimicrobials in feedlot production (Cusack & Mahony 2016). Professionals and other stakeholders need to work together to define methods to reduce antimicrobial use in animal production in South Africa. Although many students believed that new antimicrobials will be available in the future, recent trends in drug development contradict this perception. This should increase the pressure for the prudent use of currently available antimicrobials (Conly & Johnston 2005).

Of note in this study was the knowledge gap on antimicrobials among students. Half of the respondents were unsure of the pharmacodynamics, side effects and replacement alternatives for antimicrobials if first choice therapy failed, and a third of the respondents were not sure of the mechanisms of resistance of antimicrobials. It should be emphasised that these students acknowledged having received lectures on the rational use of antimicrobials and had sought additional information from other reliable sources. While an evaluation of the curriculum was beyond the scope of this study, our data suggested a perceived need for improved teaching and learning techniques on the concepts of antimicrobial use and prescriptions, including the need for recall and utilisation of knowledge of antimicrobials. Perhaps a longitudinal evaluation of a set of students in the fifth year with a re-evaluation at the end of their sixth year would have produced a different outcome and should be considered for the future. A previous study in the United Kingdom had highlighted medical students’ desires for focused antimicrobial prescription education (Heaton et al. 2008). In response to similar concerns in the United States, the Michigan State University, University of Minnesota and the Centers for Disease Control and Prevention (CDC) earlier jointly developed an open access online learning tool for veterinary students (Gordoncillo et al. 2011). The adoption of such innovative self-learning tools improves learning and promotes prudent administration of antimicrobials with consequent confidence building among the aspiring veterinarians. Furthermore, because the university conducts a periodic review of the teaching curriculum, such an exercise should consider the inclusion of targeted improvement in awareness, appropriate stewardship and prescription of antimicrobials. Faculties in the United Kingdom, for example, have made efforts to carry out regular curricula review exercises over the last decade across medical schools to enhance antimicrobial education (Davenport et al. 2005; Ross & Loke 2009).

It is noteworthy that 97% of the students agreed that an antibiogram is important as a precursor for antimicrobial therapeutics. Only 19% believed that costs should drive treatment, yet over 75% of the students acknowledged that financial constraint on the part of animal owners was a limitation to regular use of bacterial cultures and antibiotic sensitivity testing prior to treatment. It becomes important to adopt innovative and affordable methods of carrying out targeted treatment based on empirical pre-treatment evaluations. Professionals should continue to discourage affordability-induced treatment options only, a situation that may encourage over-dependence on broad-spectrum antibiotics because they are effective against a wide range of bacteria, protozoans and parasites, but may at the same time encourage resistance.

In this study, tetracycline, penicillins (particularly intra-mammary) and sulphonamides were perceived to be the most abused antimicrobials in descending order. These antimicrobial are mostly licensed for use in food animals (Fertilizers, Farm Feeds, Agricultural Remedies and Stock Remedies Act, 1947 [Act 36 of 1947], South Africa) and our findings supported a previous study on the sales volume of antimicrobials in South Africa where tetracyclines, sulphonamides and penicillin were the second-, third- and fourth- most consumed antimicrobials, respectively, in the livestock industry (Eagar et al. 2012). It should be noted that despite the perception above, many classes of antimicrobials are still strictly controlled and are only available as prescription medicines for veterinary use by law in South Africa. A number of factors including broad-spectrum activities and affordability may be responsible for the high consumption and abuse of these drugs indicated in this study. Aside from their use in extensive management of infections of anaplasma, erhlichia, theileriosis and mycoplasma, tetracycline analogues may be administered in animal feeds as growth promoters (Eagar et al. 2012). The low dosage inclusion of antimicrobials in feed for extended periods of time encourages the emergence of AMR genes, a phenomenon common in tetracyclines. Additionally, tetracyclines may be administered as a once-off parenteral dosage in addition to penicillin, which is useful in the management of Gram-positive and anaerobic bacteria (Prescott 2000; Sykes & Papich 2014). Similarly, sulphonamides are broad-spectrum antimicrobials widely applicable in poultry husbandry for the management of coccidiosis and in large animals for the treatment of calf scours and pneumonia (Mitema et al. 2001).

Most of the findings in this study are consistent with similar studies and support the conclusion that knowledge gaps currently exist on antimicrobial use in real-life situations among veterinary students (Abbo et al. 2013; Minen et al. 2010). Hence, while this study was purely exploratory and investigative, it may modify future students’ focused and targeted antimicrobial prescription and stewardship training. This survey may serve as a template to create such future-targeted training to address areas where weaknesses have been observed in this study.
